# Overexpression of OTU domain-containing ubiquitin aldehyde-binding protein 1 exacerbates colorectal cancer malignancy by inhibiting protein degradation of β-Catenin via Ubiquitin-proteasome pathway

**DOI:** 10.1080/21655979.2022.2057897

**Published:** 2022-03-30

**Authors:** Daoxiong Ye, Sisi Wang, Xiaojie Wang, Yu Lin, Ying Huang, Pan Chi

**Affiliations:** Department of General Surgery, Fujian Medical University Union Hospital, Fuzhou, Fujian Province, China

**Keywords:** OTUB1, CRC, Chemo-resistance

## Abstract

Although major advances were achieved in colorectal cancer (CRC) therapy, major concerns still remain on proper control of cancer metastasis and chemo-resistance in order to achieve satisfactory general treatment response. Previous studies suggested that OTUB1 (OTU domain-containing ubiquitin aldehyde-binding protein 1) serves as regulator of gene ubiquitination and participates in the pathogenesis of multiple malignancies. Therefore, to discover its molecular mechanism in CRC tumor growth and metastasis will contribute in CRC treatment strategy development. Clinical tissues and CRC cancer cell lines were utilized to evaluate OTUB1 expression pattern. Functional tests including cellular proliferation, migration and invasion, as well as chemo-resistance, etc., were evaluated to investigate the role of OTUB1/β-catenin regulatory pathway on CRC malignant biological behaviors. Both CRC tumor tissues and CRC cell lines exhibited promoted OTUB1 expression level. Subsequent experiments further suggested that OTUB1 promoted CRC malignancy by enhancing protein stability of β-catenin, via inhibition of its protein degradation by UPP pathway, which indicated its crucial role in enhancement of CRC tumor cellular proliferative and chemo-resistant capabilities. This study reported that OTUB1 exhibited novel pro-survival and pro-metastatic function by interaction of β-Catenin via Ubiquitin-proteasome pathway. Our research indicated that OTUB1/β-Catenin regulatory axis might be potential druggable target for CRC cancer patients’ treatment.

## Graphical abstract

**Figure:**
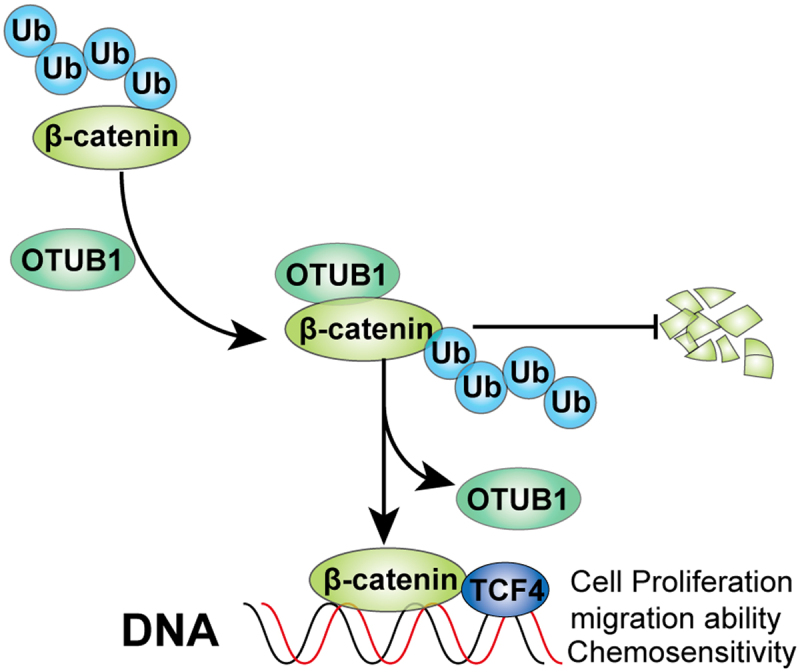

## 1. Introduction

Globally, colorectal cancer (CRC) ranks as the top-3 most common type of cancer in terms of occurrence and mortality. Despite the vast advances that have been made in the era of oncological immunotherapy, surgical operation, combined with chemotherapy, are still applied as the foundation of CRC treatment [1]. Although continuous progresses have been achieved for promoting the survival and prognosis of CRC patients, metastasis and chemo-resistance are unavoidable barriers for better general treatment response and overall prognosis. Due to the complex mechanism of CRC pathogenesis, the detailed mechanism of CRC chemo-resistance has been intensively investigated.

Multiple researches have demonstrated various dysfunctional signaling pathways, which play important roles in tumor chemo-resistance and distal organ invasion. Dysregulation of Wnt/β-Catenin signaling was proved to be involved in uncontrolled tumor expansion and differentiation inhibition [2]. It was reported that Twa1 is a previously undescribed regulator of the Wnt pathway for promoting colorectal tumorigenesis by facilitating β-catenin nuclear retention [3]. In addition, β-catenin is closely related with body mass index, and physical activity with survival in patients with colorectal cancer [4]. Several studies have also indicated that dysregulated signaling pathways including NF-κB [5], PI3K/AKT [6], RAS/RAF/MEK/MAPK [7] axis were also involved with tumor proliferation and inhibited cellular apoptosis. Besides, more regulatory networks involved in CRC pathogenesis and refractoriness are yet to be clarified. Therefore, delineation of the detailed molecular mechanism of CRC metastasis and refractoriness is of significance to develop effective strategies for metastatic disease treatment.

OTUB1 (OTU domain-containing ubiquitin aldehyde-binding protein 1) was also known as a K48 linkage-specific deubiquitinating enzyme that contains two distinct ubiquitin-binding sites [8]. OTUB1 functions as regulator of K63Ub (K63-linked polyubiquitin) synthesis in a non-canonical manner [9]. And K63Ub chain synthesis by ubiquitin-conjugating enzyme UBC13 is a vital step to repair protein recruitment to chromatin once DNA breaks form [10]. OTUB1 has been suggested to participate in the occurrence of multiple malignant diseases. However, it is still not very clear the exact role of OTUB1 in CRC tumor formation and progression. We are also intrigued about the potential impact of ubiquitin-proteasome pathway (UPP) modulation of OTUB1 on the dysregulated signal pathway involved in CRC such as Wnt/β-Catenin.

In the present study, we suspected that OTUB1 might regulate CRC through targeting β-Catenin. We aimed to find the potential regulatory mechanism for how OTUB1 affect CRC via β-Catenin. We further delineated the OTUB1ʹs impact on Wnt/β-Catenin pathway, which might provide novel insight for future CRC treatment.

## 2. Material and methods

### 2.1. Patients and sample collection

CRC cancer patients diagnosed between 5/1/2020 to 11/1/2021 were retrospectively recruited in this study. Before sample collection, patients did not receive any prior surgery, radiotherapy or chemo-therapeutic agents. Post-surgical CRC tumor tissues and matched normal sample tissues were correspondingly gathered. Harvested samples were saved in lab for subsequent experiments. The study has been approved by the Ethics Committee of Fujian Medical University Union Hospital (No. 2019–65, approval date: 2019/10/7). Declaration of Helsinki was compliant in this research. Informed consents from the patients enrolled were obtained.

### 2.2. RNA extraction and qRT-PCR experiments

RNA was extracted from cells and tissues by TRIzol agent (Invitrogen, Carlsbad, USA). RT-PCR kit (Invitrogen, Carlsbad, California, USA) was used according to the protocol for reverse-transcription. qRT-PCR was conducted utilizing primers listed in supplementary Table 1. Quantification results were further normalized to the level of GAPDH

### 2.3. Cell line culturing

SW620, HCT116, SW480, RKO, LOVO, DLD1, FHC cells were saved in our research center. Cells were cultured in DMEM medium containing 10% fetal bovine serum in an incubator with 5% CO_2_.

### 2.4. Western blotting

Western blot procedure was performed as described previously. The proteins were extracted using 1% PMSF and RIPA lysine buffers. SDS-polyacrylamide gels were used for lysates separation. Immunoblot assay was conducted with primary antibody against β-actin, β-catenin, CD44, CD133, OTUB1 (Abcam, Cambridge, MA, USA) overnight at 4°C. After washing with PBS, the proteins were incubated with second antibodies for 2 h at room temperature. ECL chemiluminescence kit (Beijing Dingguo Changsheng Biotechnology Co., Ltd, China) was used to detect protein. The protein gray was analyzed using Image J.

### 2.5. Cellular growth test

For the cellular proliferation experiment, 2000 cells per group were seeded. After vector transfection, absorbance at 490 nm was evaluated. For subsequent assay, 3000 cells per group were seeded and transfected. Then, colonies were measured and repeated three times for subsequent quantification.

### 2.6. Cellular migration and wound healing assay

For Transwell study, the procedure was according to the description previously reported elsewhere. As for wound healing experiment, each group of cells were placed and monolayers were scraped. The distance between wound was tested three times. The distance between the wound was repeatedly measured after incubation for 48 h.

### 2.7. Co-immunoprecipitation (Co-IP)

After treatment, cells were washed by PBS three times, and then cell samples were lysed in IP buffer. Afterward, lysate samples were then undergone centrifuge treatment and pre-clearing. Supernatants treated by 5 μg anti-FLAG) and A/G-Sepharose bead were further incubated at 4°C environment overnight. After that, lysis buffer was utilized for beads washing for three times and sodium dodecyl sulfate (SDS) loading buffer was used for resuspension. Western blot was subsequently performed using corresponding antibody

### 2.8. Immunofluorescence staining

First, cell samples were treated by PBS for washing, and were then treated with paraformaldehyde to fix. Afterward, after permeabilization, primary antibody was used for incubation. Subsequently, cells were incubated with conjugated secondary antibody. Then, the cells samples were stained by DAPI and examined by confocal microscopy for image generation

### 2.9. Ubiquitination test

Cell samples were treated by MG132 (Selleck Chemicals, Houston, TX, USA) after 5 h of transfection. Then, cellular lysate was treated by lysis buffer and after treatment, each cell group proteins were immune-precipitated (IP) with corresponding antibodies. Each group of cell proteins were determined and measured by Western blot.

### 2.10. Cell transfection

The target genes were amplified via PCR. The target genes were inserted into pcDNA3.1 vector. The cells were planted into a 60-mm dish. After culture in the incubator with 5% CO_2_ and 37°C for 48 h. Cell transfection was conducted using Lipofectamine 2000 (Invitrogen, US).

### 2.11. Cycloheximide (CHX)-chase test

CHX (14 μg/mL, Selleck Chemicals) was used in this study to perform CHX-chase test. CHX (14 μg/mL) was mixed with cells for 0, 2, 4, 8, respectively. Then, the protein was extracted, and protein expression levels were measured through western blotting.

### 2. 12. Statistical analysis

Statistical analysis was performed with SPSS (22.0) software. Data were harvest from three independent experiments. P < 0.05 was considered as statistical significance. T-test and ANOVA test were used for p value determination

## 3. Results

### 3.1. OTUB1 upregulation was characteristic in CRC cancer samples

**Figure 1.**
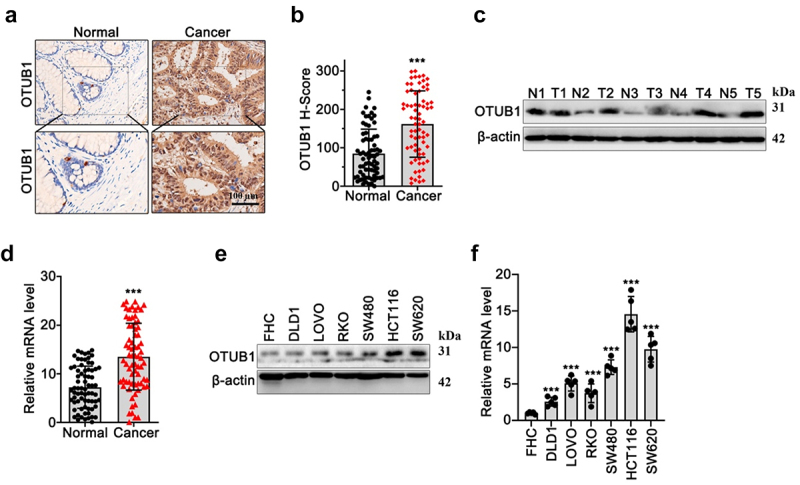
OTUB1 upregulation was found in the CRC cancer samples. (a-b) IHC staining and semi-quantitive study on OTUB1 protein expression in tumor samples of CRC patients and matched normal samples (100X). (c-d) WB/qRT-PCR investigation of OTUB1 protein and mRNA expression in CRC tumor samples and matched normal samples. (e-f) WB/qRT-PCR investigation of OTUB1 protein and mRNA expression in CRC cancer cell lines and normal mucosa cell line. *** *p* < 0.001 compared with group normal or FHC.

In our research, we investigated OTUB1 expression utilizing immunohistochemical (IHC) test on tumor and matched adjacent tissue of CRC patients. As shown in Figure 1a-b, results demonstrated that OTUB1 protein was significantly increased in tumor tissues compared with normal tissues. Moreover, WB/qRT-PCR results (Figure 1c) on CRC tumor and control normal samples (Figure 1d) showed that OTUB1 expression was notably upregulated in tumor tissues. Subsequent investigation in multiple CRC cancer and normal human mucosa cell lines indicated that OTUB1 was highly expressed in all tumor cell lines (SW620, HCT116, SW480, RKO, LOVO, DLD1), in comparison with mucosa cell line FHC (Figure 1e-f).

### 3.2. OTUB1 expression level was associated with malignant behavior of CRC cancer cells

**Figure 2.**
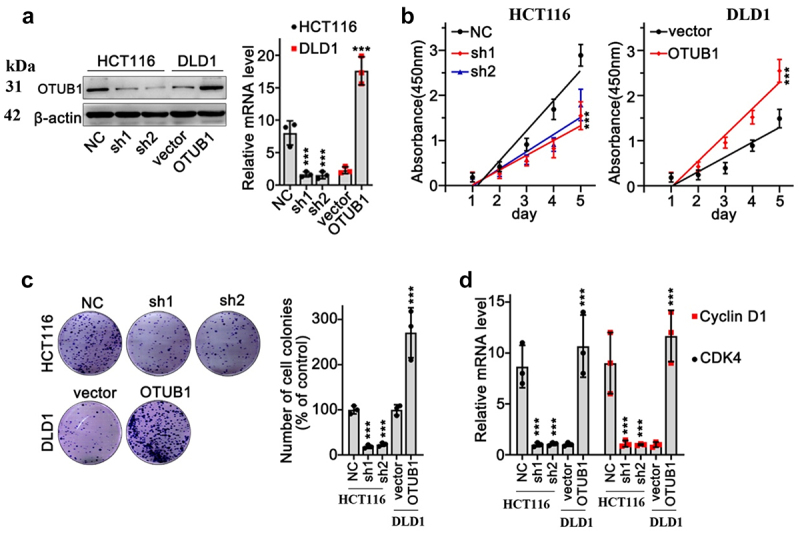
OTUB1 expression level was associated with malignant behavior of CRC cancer cells. (a) WB/qRT-PCR study on expression of OTUB1 in HCT116 and DfLD1 cell groups correspondingly treated by OTUB1-specific shRNAs and overexpression vectors. (b) Cellular proliferation test was performed in the HCT116 cells transfected with OTUB1 shRNAs and DLD1 cells transfected with OTUB1 overexpression vector. (c) Colony formation test on HCT116 and DLD1 cells correspondingly transfected with OTUB1 shRNAs and OTUB1 overexpression vector (200X). (d) qRT-PCR analysis on CDK4 and Cyclin D1 mRNA expression in HCT116 and DLD1 cell groups correspondingly transfected with OTUB1 shRNAs and OTUB1 overexpression vector. *** *p* < 0.001 compared with group NC or vector.

We aimed to detect the impact of OTUB1 modulation on CRC cancer cells. OTUB1-specific overexpression vectors and shRNA were designed and validated via qRT-PCR and WB experiments (Figure 2a-b). Then, CCK8 and colony formation tests were conducted utilizing HCT116 and DLD1 tumor cell lines. Results suggested that OTUB1 expression was associated with increased proliferation of CRC tumor cells (Figure 2b-c). Further qRT-PCR test indicated that upregulation of OTUB1 was associated with CyclinD1 and CDK4 expression elevation (Figure 2d).

### 3.3. Impact of OTUB1 overexpression in CRC tumor cell migration and invasion

**Figure 3.**
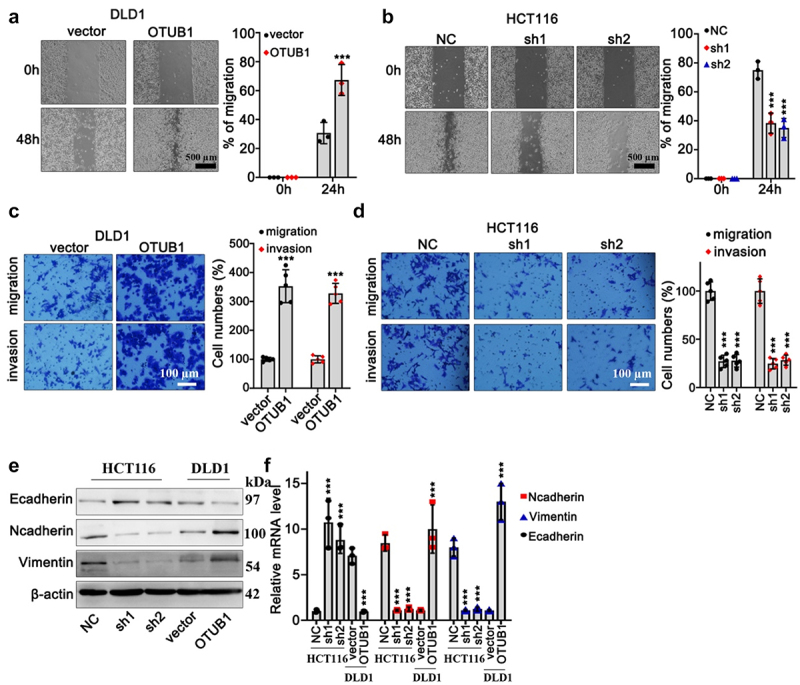
Impact of OTUB1 overexpression in CRC tumor cell migration and invasion. (a-b) Wound healing test on DLD1 and HCT116 cell line correspondingly transfected with OTUB1 overexpression vectors and specific shRNAs (100X). (c-d) Transwell test on DLD1 and HCT116 cell groups correspondingly transfected with OTUB1 overexpression vectors and specific shRNAs (400X). (e-f) WB and qRT-PCT analysis on the protein and mRNA expression of several EMT-related proteins in different cell groups correspondingly transfected with OTUB1 specific shRNAs and overexpression vectors. *** *p* < 0.001 compared with group NC or vector.

In addition, in order to further explore the impact of OTUB1 expression modulation on CRC cancer cell migrative capabilities, wound healing test on DLD1 and HCT116 cell lines were performed. OTUB1 overexpression and OTUB1-specific shRNAs were applied in each cell group. As depicted in Figure 3a, OTUB1 overexpression significantly promoted tumor cellular migrative capacity (Figure 3b). Consistently, transwell test suggested that DLD1 cells transfected with OTUB1 overexpression exhibited enhanced migration and invasion compared with control groups (Figure 3c). Meanwhile, OTUB1-specific shRNA treatment curbed HCT116 cell invasion (Figure 3d). To further investigate underlying effects of OTUB1 modulation on tumor cell epithelial-mesenchymal transition (EMT), mRNA and protein expression detection of EMT markers (E-cadherin, Vimentin, N-cadherin) indicated that OTUB1 upregulation was associated with mesenchymal phenotype (Figure 3e-f).

### 3.4. OTUB1 silencing suppressed CRC cancer cell survivability treated with multiple chemo-agents

**Figure 4.**
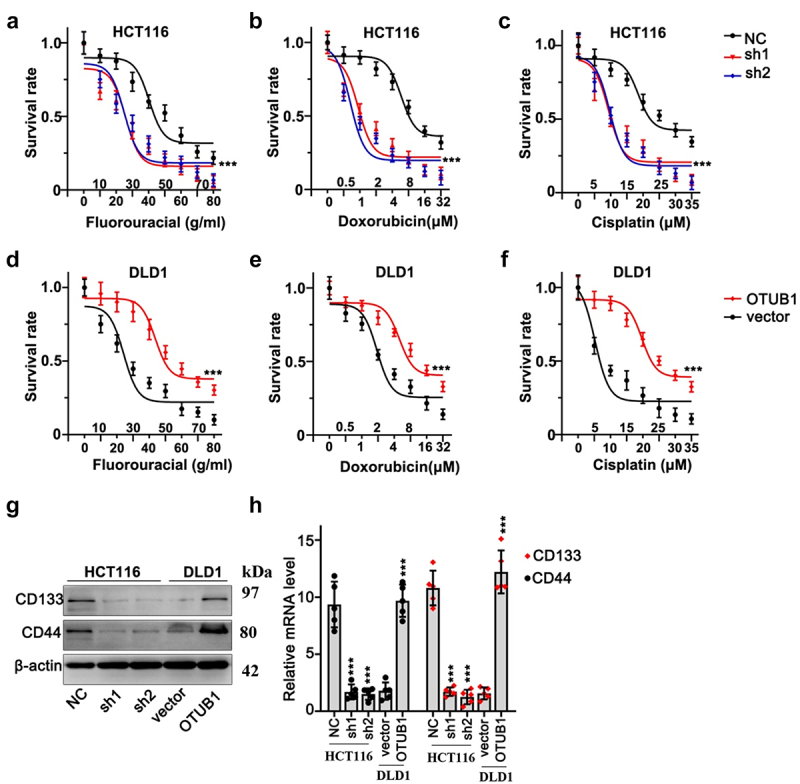
OTUB1 silencing suppressed CRC cancer cell survivability treated with multiple chemo-agents. (a-c) Cellular viability assay on HCT116 cells transfected with OTUB1 specific shRNAs. Cells were correspondingly treated by escalated dosage of fluorouracil, doxorubicin and cisplatin. (d-f) Cellular viability assay on DLD1 cell groups transfected with OTUB1 overexpression vectors. Cells were correspondingly treated by fluorouracil, doxorubicin and cisplatin under a range of dosage. (g-h) WB and qRT-PCT analysis on the protein and mRNA expression of several tumor stem-cell related genes including CD133 and CD44 in different groups correspondingly transfected with OTUB1 specific shRNAs and overexpression vectors. *** *p* < 0.001 compared with group NC or vector.

Next, we conducted cellular viability test on HCT116 and DLD1 cells with OTUB1 gene modulations. Three different chemo-agents (fluorouracil, cisplatin and doxorubicin) with multiple concentrations were utilized. As a result, OTUB1 silencing significantly suppressed HCT116 cellular survivability, while OTUB1 overexpression significantly enhanced cancer cell viabilities (Figure 4a-f). Moreover, subsequent WB and qRT-PCR test provided intriguing evidences that OTUB1 overexpression also considerably enhanced stem-cell markers of CRC tumor including CD133 and CD44 (Figure 4g-h).

### 3.5. OTUB1 exhibited tumor-promotive effects by interacting with β-Catenin and reduced its protein degradation via UPP pathway

**Figure 5.**
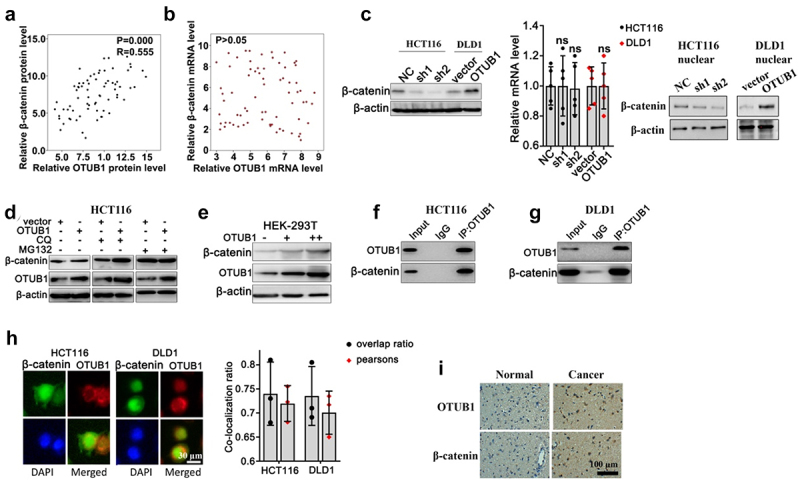
The potential impact of OTUB1 on key regulatory gene in CRC pathogenesis. (a-b) Protein and mRNA expression correlation analysis between OTUB1 and β-Catenin. (c) WB and mRNA analysis on β-Catenin protein and mRNA expression in HCT116 and DLD1 cell groups correspondingly transfected with OTUB1-specific shRNAs, OTUB1 overexpression vector and control vectors. (d) WB detection of OTUB1 and β-Catenin protein expression on HCT116 cells correspondingly transfected with OTUB1 overexpression vectors or control vectors, in combinatory treatment of two different UPP pathway inhibitors (CQ and MG132). (e) WB detection of OTUB1 and β-Catenin protein expression in HEK-293 T cell groups transfected with escalating dosage of OTUB1 overexpression vectors. (f-g) Co-IP experiment to validate the interaction between OTUB1 and β-Catenin in HCT116 and DLD1 cell lines. (h) Immunofluorescence co-localization test to detect cellular co-localization ratio of β-Catenin and OTUB1 protein expressed in HCT116 and DLD1 cell lines. (i) Immunofluorescence staining of OTUB1 and β-Catenin in normal and cancer tissues (100X).

To clarify the target of OTUB1, we focused on the potential impact of OTUB1 on key regulatory gene in CRC pathogenesis, i.e. β-Catenin. We first retrospectively evaluated the protein and mRNA expression of β-Catenin and OTUB1 in our clinical cohort, and results indicated that OTUB1 protein expression was significantly associated with protein level of β-Catenin elevated the (Figure 5a), while no significant correlation was found between mRNA level of two genes (Figure 5b). Then, WB/qRT-PCR test was performed on HCT116 and DLD1 cell groups correspondingly transfected with OTUB1 shRNAs or OTUB1 overexpression vectors. Cells transfected with OTUB1 shRNAs presented significantly inhibited β-Catenin protein expression. No remarkable changes on β-Catenin mRNA expression were detected (Figure 5c). We further utilized autophagy inhibitor (CQ) and proteasome pathway inhibitors (MG132) to investigate the role of autophagic pathway and ubiquitin-proteasome pathway (UPP) in the regulation of β-Catenin protein expression. As shown in Figure 5d, OTUB1 overexpression leaded to β-Catenin protein level increase in cell treated with CQ. However, the promotive influences were abrogated by MG132 suggesting that OTUB1 overexpression regulated β-Catenin protein expression probably through UPP. Additionally, by transfection OTUB1 overexpression vector into HEK-293 T cell line, β-Catenin protein exhibited significant increase in dose-dependent manner, which depended on the expression of OTUB1 (Figure 5e). Besides, Co-IP test as well as IF co-localization test were subsequently performed in HCT116 and DLD1 cell lines and results also confirmed direct interaction between two proteins (Figure 5f-h). Significant higher expression of OTUB1 and β-Catenin was observed in the tumor tissues (Figure 5i).

**Figure 6.**
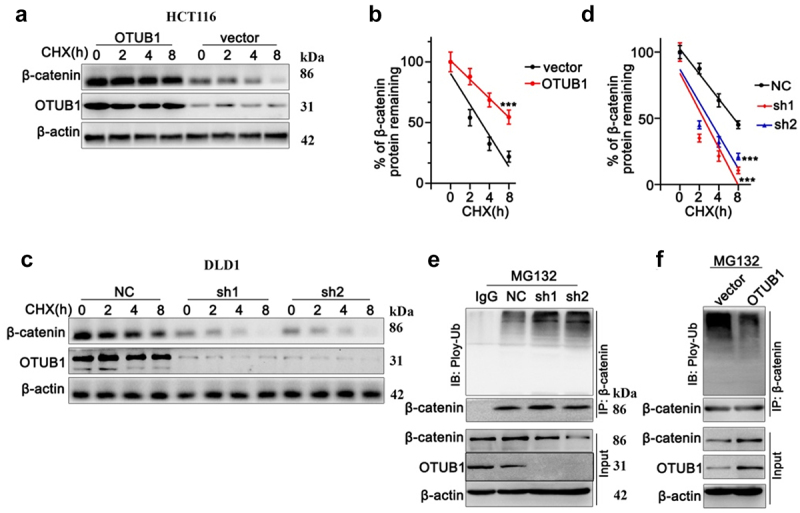
CHX chase test to detect protein degradation of OTUB1 and β-Catenin in HCT116 and DLD1 cell lines. (a-b) CHX chase test to measure OTUB1 and β-Catenin protein expression level in HCT116 cells treated with CHX for different timespan (0 h-8 h). (c-d) CHX chase test to measure OTUB1 and β-Catenin protein expression level in DLD1 cells treated with CHX for different timespan (0 h-8 h). (e) Co-Immunoprecipitation test to measure ubiquitylation of β-Catenin modulated by OTUB1 on HCT116 cells treated with MG132. (f) Co-Immunoprecipitation test to measure ubiquitylation of β-Catenin influenced by OTUB1 on DLD1 cells treated MG132. *** *p* < 0.001 compared with group NC or vector.

Next, we applied CHX chase to detect protein degradation of OTUB1 and β-Catenin in HCT116 cell line. As shown in Figure 6a, both OTUB1 and β-Catenin protein level declined gradually with CHX treatment. Notably, when transfected with OTUB1 overexpression vector, protein degradation of β-Catenin was markedly inhibited (Figure 6b). On the other hand, when transfected with OTUB1 shRNAs, the degradation of β-Catenin was significantly promoted (Figure 6c-d). Moreover, after treatment with MG132, HCT116 cells transfected with OTUB1 overexpression vector presented markedly increased ubiquitination of β-Catenin, leading to suppressed β-Catenin protein expression (Figure 6e). In contrast, when DLD1 cells were transfected with OTUB1 overexpression vectors, ubiquitination of β-Catenin was remarkably suppressed and β-Catenin protein expression presented remarkable increase (Figure 6f).

**Figure 7.**
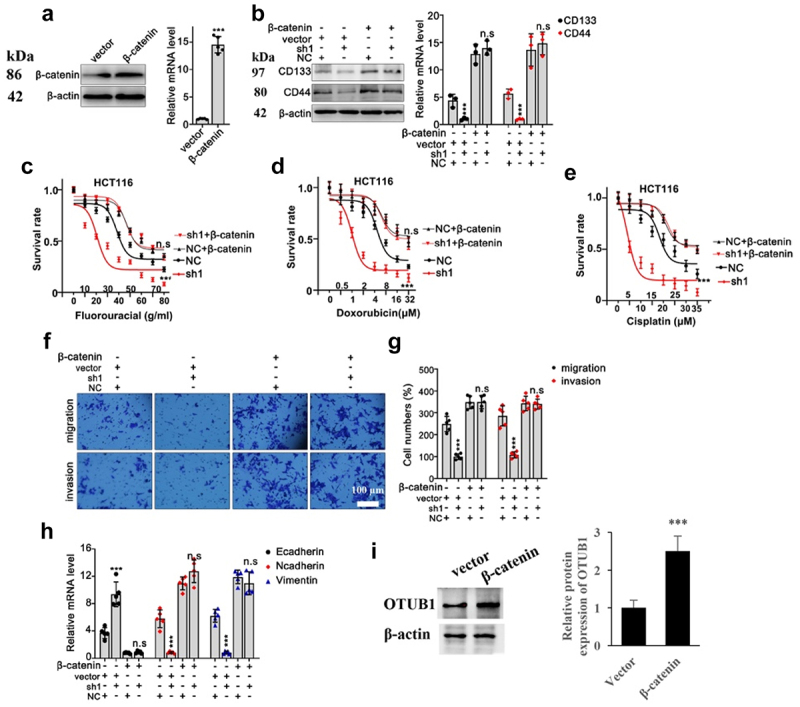
Overexpression of β-Catenin reversed the influence of OTUB1 shRNA1. (a) Design and validation of modulative effects of β-Catenin overexpression vector on β-Catenin expression in HCT116 cell line. (b) WB and qRT-PCT analysis on the protein and mRNA expression of several tumor stem-cell related genes including CD133 and CD44 in different cells correspondingly transfected with OTUB1 specific shRNAs with or without simultaneous transfection of β-Catenin overexpression vectors. (c-e) Cellular viability test on HCT116 cell groups correspondingly treated by escalated dosage of fluorouracil, doxorubicin and cisplatin. Each cell group was correspondingly transfected with β-Catenin overexpression vectors, in combination of OTUB1 specific shRNAs. (f-g) Transwell test to evaluate HCT116 tumor cell migration and invasion capabilities, each cell group was correspondingly transfected with OTUB1 specific shRNAs, with or without combinatory transfection of β-Catenin overexpression vectors (400X). (h) mRNA expression detection of several genes related with tumor cell EMT process in HCT116 cell lines. Each cell group was correspondingly transfected with OTUB1 specific shRNAs, with or without combinatory transfection of β-Catenin overexpression vectors. (i) Influence of β-Catenin overexpression vector on OTUB1 expression. *** *p* < 0.001 compared with group NC or vector.

In the following research, we further validated the above-mentioned findings by designing β-Catenin specific overexpression vector (Figure 7a). Through combinatory transfection of β-Catenin with or without OTUB1 shRNA1, we discovered that protein and mRNA expression of CD44 and CD133 were drastically suppressed in cells transfected with OTUB1 shRNA1, while the suppressive effects were abrogated through combinatory β-Catenin overexpression vector transfection (Figure 7b). Subsequent tumor viability test provided evidences that OTUB1 shRNA1 significantly suppressed HCT116 tumor cell viability under treatment of chemo-agents, while combinatory transfection of β-Catenin overexpression vector obviously reversed the suppressive effects of shRNAs (Figure 7c-e). Moreover, subsequent Transwell test also indicated that OTUB1 shRNA1 treatment hindered cellular migrative and invasive capabilities, while co-transfection with β-Catenin overexpression vector completely eliminated the suppressive effects from OTUB1 shRNA1 (Figure 7f-g). In addition, OTUB1 shRNA1 significantly suppressed Vimentin/N-Cadherin mRNA level and considerably promoted E-Cadherin mRNA level, but the impact was abolished when β-Catenin vector was co-transfected (Figure 7h). β-Catenin overexpression vector significantly promoted the OTUB1 protein expression (Figure 7i).

## 4. Discussion

In this research, we discovered that OTUB1 upregulation was typical in CRC cancer cells. And OTUB1 exhibited multi-facet promotive effects on malignant biological behaviors of CRC cancer cell including tumor cell stemness, invasion and chemo-resistance. Meanwhile, OTUB1 as de-ubiquitinase was up-regulated in several human malignancies and OTUB1 participated in tumor pathogenesis via several different routes [11,12]. For instance, previous study indicated that OTUB1 directly interacted with SLC7A11, which facilitated CD44-mediated effects on ferroptosis in human cancers [13]. Moreover, OTUB1 also participated in promotion of PD-L1 stability via hindering the degradation of PD-L1 through the ERAD pathway in tumor cells and subsequently enhanced cancer immunosuppression [14]. Besides, OTUB1 has also been demonstrated as regulator of multiple genes involving in including CyclinE1 and RAS, etc. [11,15]. The results of our study added novel evidences to the complex downstream targets of OTUB1, as we demonstrated that OTUB1 promoted β-Catenin expression by suppression its protein degradation via UPP pathway.

β-Catenin has been extensively investigated as fundamental member of Wnt/β-Catenin canonical pathway. β-Catenin participates in several vital physiological processes ranging from embryo development to adult homeostasis. Cytoplasmic β-catenin is modulated by the destruction complex containing Adenomatous polyposis coli (APC) for final degradation [16,17]. Multiple genes have been shown to cause abnormal stabilization and constitutive activation of β-catenin protein which promoted oncogenesis. Among them, loss-of-function mutations of APC were shown to contribute to the pathogenesis of CRCs via oncogenicity enhancement of downstream driver gene MYC, which subsequently caused activation of Wnt/β-catenin signaling [18]. Besides, other regulators involving chromatin remodeling, including histone methyltransferase (HMT), histone deacetylase (HDAC), and histone acetyltransferase (HAT) have also been suggested to modulate β-catenin activity [19]. OTUB1-induced β-catenin stabilization by inhibition of UPP pathway-induced protein degradation further expanded the regulatory network of Wnt/β-catenin signaling pathway. As we provided novel clues that OTUB1- β-catenin interaction resulted in CRC progression in this study, such interaction might enlighten new therapeutic target for CRC treatment strategy development in future. Strategies including direct reduction of OTUB1 expression by gene silencing or re-boosting of β-catenin degradation via UPP pathway deserve future investigation.

However, it is notable that several limitations exist in our research. As our clinical observation was based on limited-scale retrospective clinical samples, future studies with more-expanded cohorts are warranted to consolidate our findings in CRC patients. Besides, we generally detected the interaction between OTUB1 and β-catenin by cell line models. Future researches based on *in vitro and in vivo* models with OTUB1 knock-out phenotype are of great value for further exploration.

## 5. Conclusion

In this article, we proved that OTUB1 expression up-regulation was characteristic in tumor cells of CRC cancer patients. OTUB1 exacerbated CRC malignancy by promoting the protein stability β-Catenin through inhibition of UPP pathway induced β-Catenin protein degradation. Our study offers novel clues of OTUB1ʹs role in CRC oncogenesis, and enlightens future study on targeted therapy development for CRC patients.

## Supplementary Material

Supplemental Material

## References

[1] De Rosa M, Pace U, Rega D, et al. Genetics, diagnosis and management of colorectal cancer (Review). Oncol Rep. 2015;34:1087–1096. 26151224 10.3892/or.2015.4108 PMC4530899

[2] Dejana E. The role of wnt signaling in physiological and pathological angiogenesis. Circ Res. 2010;107:943–952. 20947863 10.1161/CIRCRESAHA.110.223750

[3] Lu Y, Xie S, Zhang W, et al. Twa1/Gid8 is a beta-catenin nuclear retention factor in Wnt signaling and colorectal tumorigenesis. Cell Res. 2017;27:1422–1440. 28829046 10.1038/cr.2017.107 PMC5717399

[4] Morikawa T, Kuchiba A, Yamauchi M, et al. Association of CTNNB1 (beta-catenin) alterations, body mass index, and physical activity with survival in patients with colorectal cancer. JAMA. 2011;305:1685–1694. 21521850 10.1001/jama.2011.513 PMC3087286

[5] Patel M, Horgan PG, McMillan DC, et al. NF-kappaB pathways in the development and progression of colorectal cancer. Transl Res. 2018;197:43–56. 29550444 10.1016/j.trsl.2018.02.002

[6] Narayanankutty A. PI3K/ Akt/ mTOR pathway as a therapeutic target for colorectal cancer: a review of preclinical and clinical evidence. Curr Drug Targets. 2019;20:1217–1226. 31215384 10.2174/1389450120666190618123846

[7] Scaltriti M, Baselga J. The epidermal growth factor receptor pathway: a model for targeted therapy. Clin Cancer Res. 2006;12:5268–5272. 17000658 10.1158/1078-0432.CCR-05-1554

[8] Wang T, Yin L, Cooper EM, et al. Evidence for bidentate substrate binding as the basis for the K48 linkage specificity of otubain 1. J Mol Biol. 2009;386:1011–1023. 19211026 10.1016/j.jmb.2008.12.085 PMC2682458

[9] Nakada S, Tai I, Panier S, et al. Non-canonical inhibition of DNA damage-dependent ubiquitination by OTUB1. Nature. 2010;466:941–946. 20725033 10.1038/nature09297

[10] Deng L, Wang C, Spencer E, et al. Activation of the IkappaB kinase complex by TRAF6 requires a dimeric ubiquitin-conjugating enzyme complex and a unique polyubiquitin chain. Cell. 2000;103:351–361. 11057907 10.1016/s0092-8674(00)00126-4

[11] Baietti MF, Simicek M, Abbasi Asbagh L, et al. OTUB1 triggers lung cancer development by inhibiting RAS monoubiquitination. EMBO Mol Med. 2016;8:288–303. 26881969 10.15252/emmm.201505972 PMC4772950

[12] Liao Y, Yang M, Wang K, et al. Deubiquitinating enzyme OTUB1 in immunity and cancer: good player or bad actor? Cancer Lett. 2022;526:248–258. 34875341 10.1016/j.canlet.2021.12.002

[13] Liu T, Jiang L, Tavana O, et al. The deubiquitylase OTUB1 mediates ferroptosis via stabilization of SLC7A11. Cancer Res. 2019;79:1913–1924. 30709928 10.1158/0008-5472.CAN-18-3037 PMC6467774

[14] Zhu D, Xu R, Huang X, et al. Deubiquitinating enzyme OTUB1 promotes cancer cell immunosuppression via preventing ER-associated degradation of immune checkpoint protein PD-L1. Cell Death Differ. 2021;28:1773–1789. 33328570 10.1038/s41418-020-00700-z PMC8184985

[15] Liao Y, Wu N, Wang K, et al. OTUB1 promotes progression and proliferation of prostate cancer via deubiquitinating and stabling cyclin E1. Front Cell Dev Biol. 2020;8:617758. 33537306 10.3389/fcell.2020.617758 PMC7848094

[16] Hart M, Concordet JP, Lassot I, et al. The F-box protein beta-TrCP associates with phosphorylated beta-catenin and regulates its activity in the cell. Curr Biol. 1999;9:207–210. 10074433 10.1016/s0960-9822(99)80091-8

[17] Liu C, Li Y, Semenov M, et al. Control of beta-catenin phosphorylation/degradation by a dual-kinase mechanism. Cell. 2002;108:837–847. 11955436 10.1016/s0092-8674(02)00685-2

[18] Sansom OJ, Meniel VS, Muncan V, et al. Myc deletion rescues Apc deficiency in the small intestine. Nature. 2007;446:676–679. 17377531 10.1038/nature05674

[19] Bian J, Dannappel M, Wan C, et al. Transcriptional regulation of wnt/beta-catenin pathway in colorectal cancer. Cells. 2020;9:1–29. 10.3390/cells9092125 PMC7564852 32961708

