# Supplementary material for: Overexpression of OTU domain-containing ubiquitin aldehyde-binding protein 1 exacerbates colorectal cancer malignancy by inhibiting protein degradation of β-Catenin via Ubiquitin-proteasome pathway
Source: Bioengineered. 2022 Mar 30;13(4):9106–16. doi: 10.1080/21655979.2022.2057897 (PMC9161894; doi:10.1080/21655979.2022.2057897)
Supplement: Supplemental Material [file KBIE_A_2057897_SM3990.docx]

**Supplementary Table 1** Primers used in this research

| Gene | Forward/Reverse | Sequence |
| --- | --- | --- |
| OTUB1 | Forward | 5’- GCTGGATGACAGCAAGGAGTTG |
| OTUB1 | Reverse | 5’- CTTCTCCACCTGCTCAATCAGG |
| β-Catenin | Forward | 5’- CACAAGCAGAGTGCTGAAGGTG |
| β-Catenin | Reverse | 5’- GATTCCTGAGAGTCCAAAGACAG |
| CyclinD1 | Forward | 5’- CTGGCCATGAACTACCTGGA |
| CyclinD1 | Reverse | 5’- GTCACACTTGATCACTCTGG |
| CDK4 | Forward | 5’-CCATCAGCACAGTTCGTGAGGT |
| CDK4 | Reverse | 5’-TCAGTTCGGGATGTGGCACAGA |
| CD133 | Forward | 5’- CACTACCAAGGACAAGGCGTTC |
| CD133 | Reverse | 5’- CAACGCCTCTTTGGTCTCCTTG |
| CD44 | Forward | 5’- CCAGAAGGAACAGTGGTTTGGC |
| CD44 | Reverse | 5’- ACTGTCCTCTGGGCTTGGTGTT |
| E-Cadherin | Forward | 5’- GCCTCCTGAAAAGAGAGTGGAAG |
| E-Cadherin | Reverse | 5’- TGGCAGTGTCTCTCCAAATCCG |
| N-Cadherin | Forward | 5’- CCTCCAGAGTTTACTGCCATGAC |
| N-Cadherin | Reverse | 5’- GTAGGATCTCCGCCACTGATTC |
| Vimentin | Forward | 5’- AGGCAAAGCAGGAGTCCACTGA |
| Vimentin | Reverse | 5’ - ATCTGGCGTTCCAGGGACTCAT |
| GAPDH | Forward | 5’ -GTCCATGCCATCACTGCCAC |
| GAPDH | Reverse | 5’ -AAGGCTGTGGGCAAGGTCAT |
